# Allopurinol-Induced Drug Reaction With Eosinophilia and Systemic Symptoms (DRESS) Syndrome: A Case Report and Review of the Published Evidence From India

**DOI:** 10.7759/cureus.113430

**Published:** 2026-07-27

**Authors:** Anjali Shinde, Vandana M Thorat, Varsha Jamale, Smita D Gaikwad, Bijoy Panda

**Affiliations:** 1 Pharmacology, Krishna Institute of Medical Sciences, Krishna Vishwa Vidyapeeth (Deemed to be University), Karad, IND; 2 Dermatology, Krishna Institute of Medical Sciences, Krishna Vishwa Vidyapeeth (Deemed to be University), Karad, IND; 3 Pharmacology, Krishna Charitable Hospital and Medical Research Center, Krishna Vishwa Vidyapeeth (Deemed to be University), Karad, IND; 4 Pharmacy Practice, Krishna Institute of Pharmacy, Krishna Vishwa Vidyapeeth (Deemed to be University), Karad, IND

**Keywords:** allopurinol, dress syndrome, drug hypersensitivity syndrome, eosinophilia, severe cutaneous adverse reactions (scars)

## Abstract

We report a case of allopurinol-induced drug reaction with eosinophilia and systemic symptoms (DRESS) syndrome in a 58-year-old hypertensive female who developed generalized erythematous, pruritic skin lesions approximately 35 days after initiating allopurinol (100 mg twice daily) for a provisional diagnosis of gout. Dermatological examination revealed diffuse erythematous to hyperpigmented scaly plaques involving the face, trunk, groin, buttocks, and extremities. Laboratory investigations demonstrated marked eosinophilia (30%; absolute eosinophil count of 880 cells/mm³), persistent leukocytosis, transient thrombocytopenia (140,000/mm³), acute kidney injury with elevated blood urea (102-116 mg/dL) and serum creatinine (1.8-3.8 mg/dL), and hepatic dysfunction with elevated alanine aminotransferase (130 IU/L) and alkaline phosphatase (179 U/L). Infectious and autoimmune causes were excluded, and a RegiSCAR score of 7 established a definite diagnosis of DRESS syndrome. Allopurinol was immediately discontinued, and the patient was treated with intravenous hydration, systemic corticosteroids, antihistamines, and supportive care, followed by a gradual tapering of prednisolone. The patient showed complete clinical recovery without recurrence. This case highlights the importance of early recognition of allopurinol-induced DRESS syndrome, prompt withdrawal of the offending drug, timely initiation of systemic corticosteroid therapy, and close monitoring of renal and hepatic involvement to achieve favorable clinical outcomes. It also underscores the value of pharmacovigilance and adverse drug reaction reporting in improving medication safety.

## Introduction

Drug reaction with eosinophilia and systemic symptoms (DRESS) syndrome, also known as drug-induced hypersensitivity syndrome (DIHS), is a rare but potentially life-threatening severe cutaneous adverse reaction characterized by widespread rash, fever, eosinophilia, and multiorgan involvement. It occurs in approximately 1 in 1,000-10,000 drug exposures and has a mortality rate of 10%-20% [1-4]. Allopurinol, a xanthine oxidase inhibitor and a first-line urate-lowering agent for the management of gout and hyperuricemia, is widely prescribed in India because of its affordability, availability, and inclusion in clinical practice guidelines. It is a globally well-recognized cause of severe cutaneous adverse reactions (SCARs), including DRESS syndrome, Stevens-Johnson syndrome (SJS), and toxic epidermal necrolysis (TEN) [5,6]. Despite its widespread use, published Indian case reports of allopurinol-induced DRESS syndrome remain limited, resulting in a paucity of population-specific data.

## Case presentation

A 58-year-old female with a known history of hypertension for the past eight years, managed with telmisartan and hydrochlorothiazide, was prescribed oral allopurinol 100 mg twice daily at an outside clinic for a suspected diagnosis of gout. She presented to the dermatology department with an eight-day history of progressively worsening generalized erythematous skin lesions associated with severe pruritus, a burning sensation, and a bitter taste in the mouth. The patient reported an episode of fever and chills preceding the onset of the rash. The cutaneous lesions initially appeared and subsequently spread symmetrically to involve the face, chest, abdomen, back, bilateral upper and lower limbs, groin, and buttocks. Approximately 35 days before the onset of symptoms, she had been started on allopurinol 100 mg twice daily along with several concomitant medications, including tramadol-paracetamol, Livogen, febuxostat, Mahayograj Guggul, and amitriptyline. There was no history of recent infection, psychological stress, atopy, angioedema, diabetes mellitus, tuberculosis, COVID-19 infection, or previous similar episodes, and the family history was non-contributory. On examination, the patient was afebrile, with a blood pressure of 120/80 mmHg, pulse rate of 82 beats/min, respiratory rate of 22 breaths/min, and bilateral pitting edema. Systemic examination of the cardiovascular, respiratory, abdominal, and central nervous systems was unremarkable.

**Figure 1:**
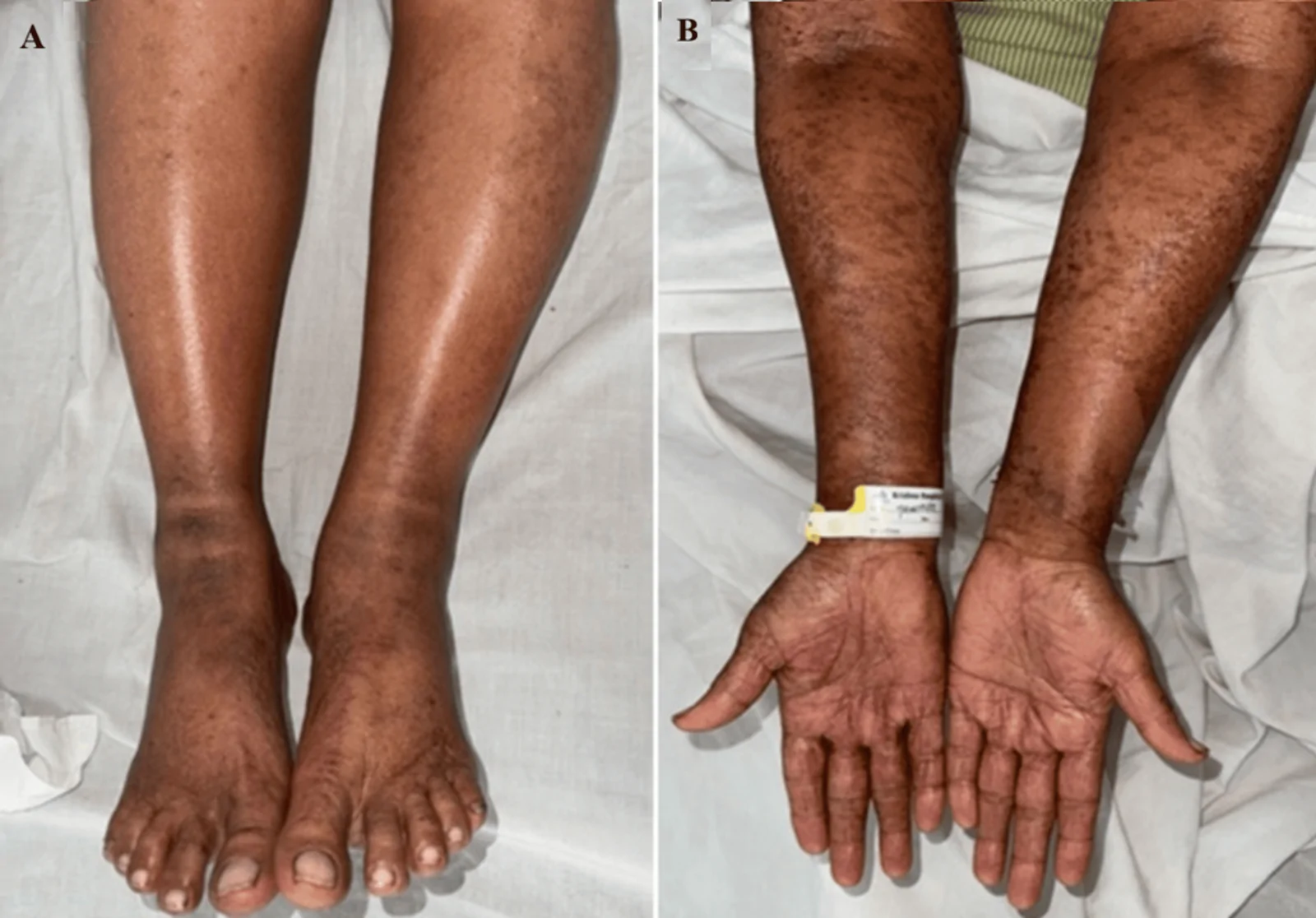
(A, B) Multiple, symmetrically distributed dark brown to black hyperpigmented macules and patches over the bilateral extremities.

Dermatological examination revealed widespread erythematous to hyperpigmented scaly plaques involving the face, chest, back, abdomen, and extremities. There were multiple, symmetrically distributed hyperpigmented macules and patches of dark brown to black coloration noted over the bilateral extremities. The lesions were diffuse, speckled, and confluent in certain areas, with associated xerosis and mild lichenification, as depicted in Figure 1A, B.

**Figure 2:**
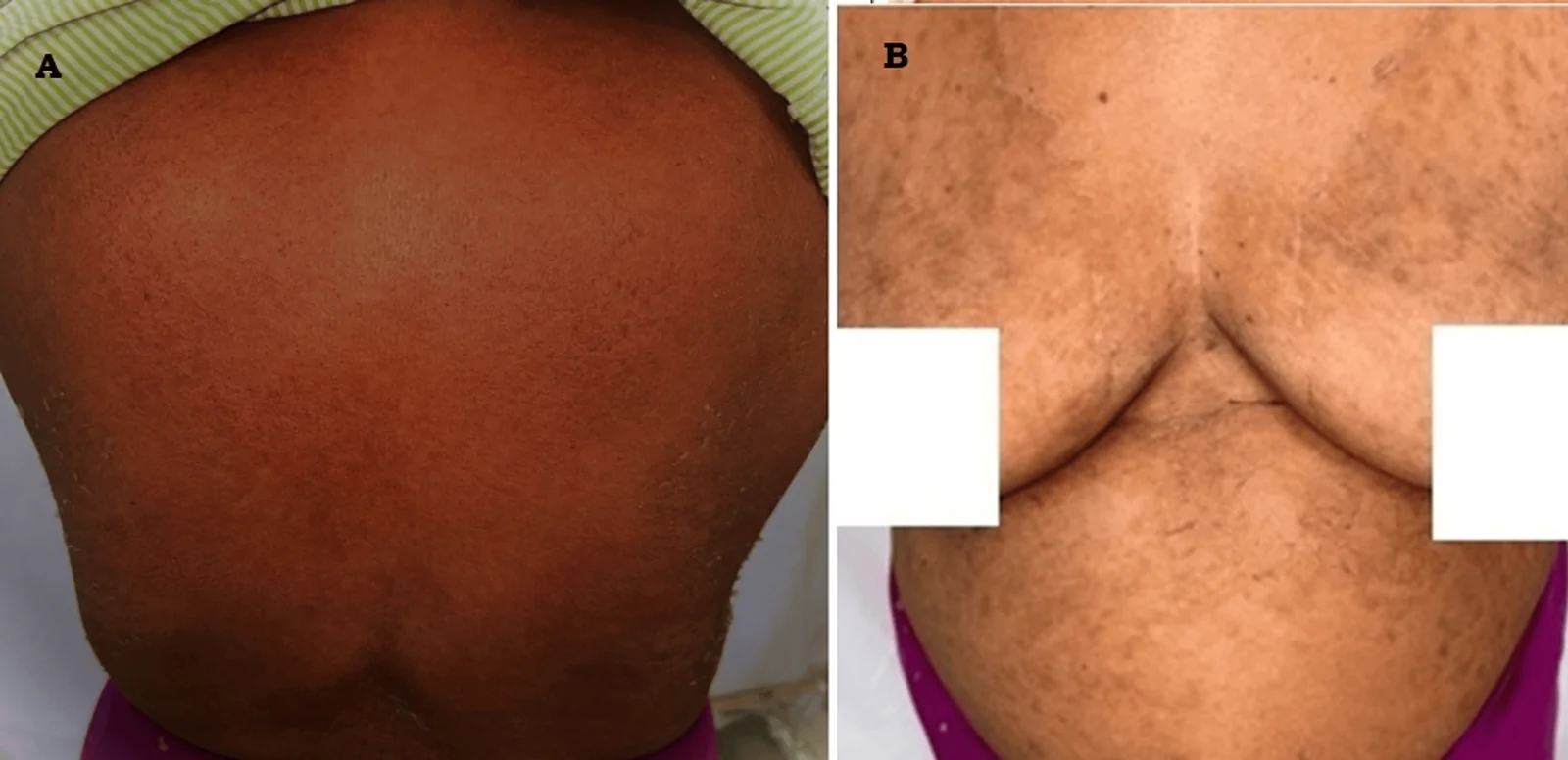
Skin changes over the trunk. (A) Posterior view; (B) frontal view.

Figure 2A shows the posterior view of the trunk demonstrating diffuse erythema with fine superficial scaling, most prominent over the flanks and lateral aspects of the back. The skin surface appears dry, with a subtle ichthyosiform texture and adherent whitish scales over the lower back region. Figure 2B shows the frontal view of the trunk demonstrating diffuse reticulate hyperpigmentation over the chest and abdomen. The skin appears mildly thickened with accentuated skin markings and scattered dark macules distributed across the torso.

Serial laboratory investigations demonstrated multisystem involvement consistent with allopurinol-induced DRESS syndrome. Hematological evaluation revealed marked eosinophilia (30%; absolute eosinophil count of 880 cells/mm³) at presentation, a hallmark feature of DRESS, along with mild leukocytosis that persisted during hospitalization. Transient thrombocytopenia (140,000/mm³) and a reduction in hemoglobin were also observed. Renal function tests showed severe acute kidney injury, evidenced by markedly elevated blood urea (102-116 mg/dL) and serum creatinine (1.8-3.8 mg/dL). Liver function tests demonstrated hepatic involvement, with elevated serum alkaline phosphatase (179 U/L) and alanine aminotransferase (130 IU/L), indicating mixed hepatocellular and cholestatic injury (Table 1).

**Table 1:** Hematological, renal, and liver function parameters during hospitalization in a patient with allopurinol-induced DRESS syndrome. DRESS: drug reaction with eosinophilia and systemic symptoms.

Parameter	Normal values	Day 1 of admission	Day 3 of admission	Day 8 of admission
Hematological investigations				
Hemoglobin (g/dL)	12.0-15.0	12.7	10.0	10.9
Total leukocyte count (cells/μL)	4,000-11,000	10,600	12,000	11,600
Neutrophils/lymphocytes/eosinophils (%)	40-70/20-40/1-6	38/30/30	82/10/06	70/25/02
Absolute eosinophil count (cells/μL)	40-440	880	-	-
Platelet count (cells/μL)	150,000–450,000	221,000	140,000	183,000
Renal function tests				
Blood urea (mg/dL)	13-43	102	116	45
Serum creatinine (mg/dL)	0.6-1.2	1.8	3.8	1.2
Liver function tests				
Serum alkaline phosphatase (U/L)	46-122	179	-	-
Alanine aminotransferase (IU/L)	5-34	130	-	98

The Registry of Severe Cutaneous Adverse Reactions (RegiSCAR) scoring criteria was applied [4], yielding a score of 7, consistent with a definite diagnosis of DRESS syndrome (Table 2). Blood culture as well as tests for hepatitis viruses (including hepatitis A, B, and C) and human immunodeficiency virus (HIV) were all negative. Antinuclear antibodies (ANA) and antistreptolysin O (ASO) titers were also found to be negative.

**Table 2:** RegiSCAR group scoring system for diagnosing DRESS syndrome in hospitalized patients. RegiSCAR: Registry of Severe Cutaneous Adverse Reactions; DRESS: drug reaction with eosinophilia and systemic symptoms.

Criterion	Reference score	Patient's score
Fever ≥38.5°C	No/unknown = 0; yes = 0	0
Enlarged lymph nodes (≥2 sites, >1 cm)	No = 0; yes = +1	0
Eosinophilia	AEC 700–1499 cells/µL or 10–19.9% = +1; AEC ≥1500 cells/µL or ≥20% = +2	+1
Atypical lymphocytes	No = 0; yes = +1	Not documented
Skin rash >50% body surface area	No = 0; yes = +1	+1
Rash suggestive of DRESS (≥2 of facial edema, infiltration, purpura, desquamation)	No = 0; yes = +1	+1
Skin biopsy suggesting another diagnosis	Yes = −1	Not documented
Internal organ involvement	One organ = +1; two or more organs = +2	+2
Resolution >15 days	No/unknown = 0; yes = +1	+1
Evaluation of ≥3 alternative causes negative	No = 0; Yes = +1	+1

Allopurinol, prescribed for a suspected gout condition, was identified as the offending agent and was immediately discontinued. No further doses were administered during hospitalization. The patient was managed with intravenous hydration using 0.9% normal saline (NS), systemic corticosteroids, an antihistamine, and supportive care. Initial treatment included intravenous hydrocortisone 100 mg and pheniramine 25 mg, followed by oral prednisolone 60 mg/day for eight days. Symptomatic management of pruritus was achieved with hydroxizine 25 mg three times daily and cetirizine 10 mg twice daily. Additional supportive therapy included gastric protection, an antiemetic as required, and close monitoring of renal and hepatic function. Prednisolone was gradually tapered following clinical improvement. The tapering regimen consisted of 60 mg/day for 15 days, followed by 50 mg/day for 15 days, 40 mg/day for 15 days, 30 mg/day for 30 days, and 20 mg/day for 15 days. Subsequently, the dose was further reduced using an alternate-day regimen before tapering to 10 mg/day and eventual discontinuation, with close clinical monitoring throughout the tapering period. The patient tolerated the taper without recurrence of symptoms or steroid withdrawal manifestations. The patient developed definite allopurinol-induced DRESS syndrome. The patient was advised to permanently discontinue allopurinol and to avoid it for life. A drug alert card was issued to the patient and the caregiver.

Febuxostat, 40 mg/day, was started only after complete clinical stabilization, with resolution of the cutaneous manifestations and significant improvement in renal and hepatic function. At the time of initiation, the patient's serum creatinine had normalized to 1.0 mg/dL, blood urea had decreased to 45 mg/dL, and liver enzymes had shown progressive improvement. However, serum uric acid remained elevated (8.3 mg/dL), warranting consideration of an alternative urate-lowering therapy. During follow-up, the patient underwent regular clinical assessment. No evidence of recurrent hypersensitivity or deterioration in renal or hepatic function was observed. 

## Discussion

This case highlights the classical clinical presentation of allopurinol-induced DRESS syndrome, characterized by the delayed onset of symptoms approximately five to six weeks after initiation of therapy, diffuse maculopapular rash with mucosal involvement, eosinophilia, hepatic dysfunction, and acute kidney injury.

To contextualize the present case, a narrative review was conducted using PubMed and Google Scholar databases to identify published Indian case reports of allopurinol-induced DRESS syndrome. The search included articles published from January 2010 to the date of the search using the predefined search terms: (allopurinol [Title/Abstract]) AND ("drug reaction with eosinophilia and systemic symptoms" [Title/Abstract] OR DRESS [Title/Abstract] OR DIHS [Title/Abstract]) AND (India [Title/Abstract] OR Indian [Title/Abstract]). Only English-language full-text case reports involving Indian patients were included, whereas review articles, editorials, conference abstracts, clinical guidelines, and reports lacking adequate clinical details were excluded. Four eligible Indian case reports were identified (Table 3). Similar to our patient, these reports demonstrated that allopurinol-induced DRESS predominantly affected middle-aged adults (28-62 years), showed a female predominance (4 of 5 cases), and commonly presented with generalized cutaneous eruptions, eosinophilia, and multiorgan involvement, particularly affecting the liver and kidneys [7-11].

**Table 3:** Comparison of published case reports of allopurinol-induced DRESS syndrome, highlighting patient demographics, allopurinol regimen, duration of therapy, clinical presentation, organ involvement, RegiSCAR score, HLA-B*58:01 testing, and clinical outcomes. DRESS: drug reaction with eosinophilia and systemic symptoms; RegiSCAR: Registry of Severe Cutaneous Adverse Reactions; CKD: chronic kidney disease.

Author [Ref]	Age/gender	Allopurinol dose regimen	Duration of therapy	Major clinical features	Organ involvement	RegiSCAR group score	HLA-B*58:01 tested	Outcome
Pawar et al. [7]	45/Female	100 mg twice daily	3 months	Pruritic rash, facial edema; skin biopsy: interface dermatitis	Liver, renal	Not reported	Not reported	Recovered
Neki et al. [8]	62/Female	100 mg once daily	15 days	High-grade fever, diffuse erythematous maculopapular rash, facial edema, generalized lymphadenopathy, and severe pruritus	Skin, liver, eosinophilia	Not reported	Not reported	Recovered
Kumar et al. [9]	52/Female	100 mg three times daily	2 weeks	Itchy erythematous maculopapular lesions over the chest; progressively spreading over the entire body. One week later, fever developed	Skin, liver, eosinophilia	Not reported	Not reported	Recovered
Karthika et al. [10]	54/Female	100 mg	3 months	Fever, generalized purpuric rash with severe pruritus involving the oral cavity, lips, upper back, and lower limbs	Eosinophilia, low hemoglobin, elevated serum urea, renal	Not reported	Not reported	Recovered
Mahato et al. [11]	Early 60/Male	100 mg once daily	3-4 weeks	Mild erythema to widespread maculopapular rash involving the chest, abdomen, trunk, and limbs, facial puffiness, pallor, and bilateral pitting pedal edema	Fever, rash, eosinophilia, hepatic dysfunction, and existing CKD leading to acute kidney injury	Performed; however, score not reported	Not reported	Recovered

These observations are consistent with findings from a recent systematic review of worldwide case reports, which reported a median patient age of 61 years and a slight male predominance. Most patients had underlying comorbidities, particularly hypertension, chronic kidney disease, diabetes mellitus, and coronary artery disease. Allopurinol was prescribed primarily for asymptomatic hyperuricemia or gout, and the median latency period between drug initiation and symptom onset was approximately 3.7 weeks, although presentations ranged from one week to several months. The global review also demonstrated that cutaneous rash, fever, eosinophilia, facial edema, and lymphadenopathy were the most common clinical manifestations, while hepatic (88%) and renal (68.7%) involvement represented the predominant internal organ complications. Similar multisystem involvement was observed in our patient, who developed marked eosinophilia, elevated liver enzymes, and acute kidney injury, all of which improved following discontinuation of allopurinol [6].

Our patient demonstrated marked eosinophilia together with leukocytosis, findings that strongly support the diagnosis of DRESS syndrome. Eosinophilia represents one of the defining hematological abnormalities included in the RegiSCAR diagnostic criteria and reflects activation of the Th2-mediated immune response with increased interleukin-5 production. The transient thrombocytopenia and mild anemia observed during hospitalization may be explained by systemic inflammation and immune-mediated hematological involvement, both of which have been reported in severe cases of DRESS syndrome. The gradual normalization of hematological parameters following discontinuation of allopurinol and corticosteroid therapy further supports an immune-mediated mechanism rather than a primary hematological disease. The predominance of hepatic and renal involvement is biologically plausible because activated drug-specific T lymphocytes and eosinophils infiltrate multiple organs following recognition of drug-derived antigens, resulting in immune-mediated tissue injury through cytokine release, cytotoxic lymphocyte activation, and eosinophilic inflammation. The simultaneous presence of elevated liver enzymes and acute kidney injury in our patient therefore reflects the characteristic multisystem nature of DRESS syndrome rather than isolated drug toxicity.

Management of our patient closely paralleled current recommendations and previously published experience. Immediate discontinuation of allopurinol represented the cornerstone of therapy, while systemic corticosteroids were administered because of significant internal organ involvement. Similar management strategies have been described in both Indian and international reports, with corticosteroids remaining the preferred first-line treatment [6-10]. 

Febuxostat can be selected because it is a non-purine selective xanthine oxidase inhibitor with a chemical structure distinct from allopurinol and is recommended as an alternative in patients with allopurinol intolerance or hypersensitivity. Although rare cases of febuxostat-associated cutaneous adverse reactions have been reported, the risk of cross-reactivity appears to be low. Therefore, febuxostat was initiated at a low dose (40 mg once daily) under close clinical supervision.

Before initiation, the patient was counseled regarding the potential risk of recurrent hypersensitivity reactions and was advised to discontinue the medication immediately and seek medical attention if symptoms such as rash, fever, mucosal lesions, or systemic manifestations developed.

This study has several strengths. In addition to describing a well-characterized contemporary case, it provides a structured synthesis of published Indian evidence using predefined search criteria, thereby offering a consolidated overview of the demographic characteristics, clinical presentation, organ involvement, management strategies, and outcomes reported from India. This approach enhances the clinical relevance of the report by placing the current case within the broader national context. This case highlights the importance of ADR reporting under the Pharmacovigilance Programme of India (PvPI) to strengthen drug safety and improve patient care. Hence, it was reported to our PvPI Adverse Drug Reaction Monitoring Centre (AMC) for submission through VigiFlow. Nevertheless, several limitations should be acknowledged. The report describes a single patient, limiting its generalizability. HLA-B*58:01 genotyping, patch testing, and lymphocyte transformation testing were not available. 

This case highlights the need for heightened clinical awareness of delayed hypersensitivity reactions associated with allopurinol and reinforces the importance of early diagnosis using the RegiSCAR criteria, prompt withdrawal of the offending drug, and multidisciplinary management. It also supports strengthening pharmacovigilance through routine reporting of severe cutaneous adverse drug reactions to the PvPI. Furthermore, the structured synthesis of Indian evidence provides a foundation for future multicenter registries, prospective studies, and the development of India-specific recommendations for pharmacogenetic screening, diagnosis, and management of allopurinol-induced DRESS syndrome.

## Conclusions

This case highlights the importance of maintaining a high index of suspicion for allopurinol-induced DRESS syndrome in patients presenting with delayed-onset cutaneous eruptions, eosinophilia, and evidence of multiorgan involvement following allopurinol therapy. Early recognition, prompt withdrawal of the offending drug, application of the RegiSCAR diagnostic criteria, and timely initiation of systemic corticosteroids were associated with favorable clinical recovery in our patient. Given the potentially life-threatening nature of DRESS syndrome, patients require careful long-term clinical follow-up, including serial monitoring of renal and hepatic function during and after corticosteroid tapering, to detect relapse or delayed autoimmune complications. Lifelong avoidance of allopurinol and appropriate patient counseling, including documentation of the allergy through an ADR alert card, are essential to prevent inadvertent re-exposure.
